# Quality of life of patients on treatment for latent tuberculosis infection: a mixed-method study in Stockholm, Sweden

**DOI:** 10.1186/s12955-019-1228-4

**Published:** 2019-10-24

**Authors:** Jad Shedrawy, Lena Jansson, Isac Röhl, Asli Kulane, Judith Bruchfeld, Knut Lönnroth

**Affiliations:** 1 Department of Public Health, Karolinska Institutet, Solnavägen 1E, Stockholm, Sweden; 2 Division of Infectious Diseases, Department of Medicine, Solna, Karolinska Institutet, Stockholm, Sweden; 3 Department of Infectious Disease, Karolinska University Hospital, Stockholm, Sweden; 4 Centre for Epidemiology and Community Medicine, Stockholm County Council, Stockholm, Sweden

**Keywords:** Health-related quality of life, Latent tuberculosis, Screening, Migrant, Mixed methods design

## Abstract

**Background:**

Unlike active tuberculosis, latent tuberculosis infection (LTBI) is asymptomatic and often considered not to affect the health-related quality of life (HRQoL) of patients. However, being diagnosed with and treated for LTBI can be associated with adverse clinical evens such side effects of treatment as well as psychosocial challenges. Therefore, the aims of this study were to qualitatively explore patients’ experiences during diagnosis and treatment of LTBI in Stockholm measure their HRQoL, and contrast and merge the results to better understand how the HRQoL of these patients is affected.

**Methods:**

LTBI patients who were treated in Stockholm during September 2017 and June 2018and who fulfilled the inclusion criteria were invited to fill a survey that included a HRQoL instrument, EQ-5D-3 L, and a mental health screening instrument, RHS-15. After filling the survey, a subset of these patients was asked to participate in an interview with open-ended questions that focused on their experiences during the diagnosis and treatment.

**Results:**

In total 108 participants filled that survey and interviews were conducted with 20 patients. Patients scored relatively high on EQ-5D: the scores of utility and VAS scale are similar to those reported by the general population of Stockholm. Very few patients reported problems on the physical health domains of EQ-5D which was supported by the quantitative data that showed no effect on physical health and usual activity. Thirty-eight percent screened positive for RHS-15 and 27.8% reported problems with anxiety/depression domain of EQ-5D which could be related to many stressing factors mentioned in the interviews such as: fear and distress related to lack of clarity about LTBI diagnosis, perceived risk of infecting others and uncertainties about the future.

**Conclusion:**

The quantified HRQoL of LTBI patients in Stockholm is similar to the general population and there is thus no HRQoL decrements that is detectable with EQ-5D. However, the study reinforces the importance of tackling anxiety and fear and ensuring good health information for persons diagnosed with and treated for LTBI.

## Introduction

Unlike the active form of the disease, latent tuberculosis infection (LTBI) is an asymptomatic condition in which patients carry the bacteria but do not show any sign of illness, however they are at risk of disease activation at any time in the future [1]. LTBI preventive treatment can reduce the risk of activation however it comes with a risk of adverse drug reactions [2]. Therefore Screening and treatment of LTBI may be recommended if the potential benefit of preventive treatment outweighs the risk of side effects [1]. LTBI management is a part of TB control mainly in low incidence countries such as Sweden where around 90% of TB cases are foreign born. National Swedish policy on TB prevention recommends systematic LTBI screening of selected migrants from high TB-incidence countries [3, 4].

Despite being asymptomatic, a diagnosis of LTBI may cause anxiety and raise concerns among patients in addition to the risk of adverse drug reactions of preventive treatment [5]. This diagnosis may also be miscomprehended as active TB which can increase stigma and psychological stress [6, 7]. All these factors can impair the Health-Related Quality of Life (HRQoL) of patients in term of physical, mental and/or social well-being.

There are few studies in the literature on HRQoL of this patient group. Studies from Canada and the United States show no statistically significant decrease of HRQoL of LTBI patients and therefore it is often considered that this patient group has similar HRQoL to the general population [8, 9]. However, these studies along with the current literature do not share any knowledge about factors influencing this HRQoL or a deep understanding of the physical and mental well-being of these patients.

Heath status and the effect of LTBI diagnosis and treatment are important as successful strategies to reduce the TB burden globally includes LTBI management especially in low incidence setting, and therefore data about HRQoL is needed to assess the effectiveness and cost-effectiveness of different interventions [10, 11]. Hence, came the rational of this mixed method study to understand the HRQoL not only quantitatively through validated instruments but also through patients interviews that capture some knowledge and dimensions that are not covered quantitatively.

The aim of this mixed-method study is to answer 3 main questions: what is the HRQoL of LTBI patients in Stockholm and what is the prevalence of mental health concerns among them? How did these patients experience the diagnosis and treatment of LTBI? And what are the factors influencing the HRQOL of these patients?

## Methods

### Study design

This cross-sectional study was conducted through surveys distributed to LTBI patients followed by interviews with a subset of them to better understand what factors influence HRQoL and to validate, contrast or explain the quantitative scores obtained through the survey.

This study follows a mixed method design building upon the strengths of both qualitative and quantitative methods [11, 12]. the sampling for the qualitative group did not depend on the quantitative results, rather some patients were asked to participate based on some sociodemographic sampling and the interviews took place at the same time as the survey; therefore this mixed method design followed the parallel convergent design time allowing the examination of both data in a concurrent fashion with an advantage of decreasing the risk of no-participation which could be suspected in this patient group if a sequential design was used instead(ex. interviews taking place in the next visit) [13, 14].

### Study population and recruitment

The study participants were patients aged 16 or older diagnosed and under treatment for LTBI at the Infectious Disease or Pediatric clinics in Karolinska University Hospital, Sweden. Patients were identified through electronic patient records and recruited consecutively between September 2017 and June 2018. The required sample size to detect a significant relative difference in HRQoL score of 9% (5% significance level and 80% power) between persons on treatment for LTBI and the general population was calculated to be 52. The 9% difference was reported in a study comparing LTBI utility scores to a control group [9]. Consecutive sampling was considered a suitable equivalent to probability sampling technique for this study and all patients that met the inclusion criteria during the study period were invited to participate in the survey [15].

LTBI Patients with disabling medical conditions that were the main indication for LTBI screening, such as systemic rheumatic diseases, dialysis, transplantation, and other conditions requiring immunosuppressive therapy were excluded. Patients with a language barrier who did not speak any of the languages in which the EQ-5D instrument was available or found it difficult to understand the questionnaire were also excluded.

The socio-demographic characteristics were assessed while conducting the survey and a sub-sample of participants were purposively invited to participate also in the qualitative interview based on their age, gender, pregnancy history and country of origin. The reasoning behind this sampling was to recruit participants so that the qualitative and quantitative samples would be comparable to a high extent. Data collection for the qualitative interviews stopped when data saturation, the point at which no new information emerged, was reached.

### Data collection

HRQoL was assessed using the validated instrument EQ-5D-3 L developed by the EuroQol Group [16]. The instrument consists of 2 main parts: (a) a descriptive system of 5 dimensions (mobility, self-care, usual activities, pain/discomfort and anxiety/depression) where the participant indicates for each dimension whether he/she has no problems, some problems or severe problems; (b) a visual analogue scale (VAS) where the participant self-rates health on a vertical visual scale from 0 (worst health) to 100 (best health) [16]. Refugee health screening-15 (RHS-15), a validated instrument of 15 questions, was used to screen for mental health issues among study subjects with a migration background [17].

The qualitative data collection was facilitated by an interview guide that was developed to capture the experiences of the patients during the diagnosis and treatment of LTBI and the factors influencing their HRQoL. Interviews were completed by JS and LJ who are fluent in either Arabic, English, Swedish or French. For interviews conducted in Tigrinja, Somali, Mongolian, Amarinja and Thai, certified trained translators were used.

### Analysis

The EQ-5D domains were dichotomized into no problems or problems (some or severe) and described as counts (%) with the 95% confidence intervals. The scores from these domains were used to generate EQ-5D utility scores. In addition, the EQ-VAS median, 25th and 75th interquartile range (IRQ) were calculated. The data from this study was compared to another EQ-5D data set representing the general population of Stockholm County which was obtained through “Hälsa Stockholm 2014” a public health survey performed by Stockholm County Council [18]. The scores from RHS-15 questions were dichotomized to either a positive or negative screening outcome as per the instrument validation, and described by the counts (%) of people with positive screening with the 95% confidence interval.

As the EQ-5D data was positively skewed, it was dichotomized into 2 groups below and above the median value. Chi-square tests were performed to test whether LTBI patients had significantly different HRQoL measures compared to the general population of Stockholm. Phi coefficients were obtained through Chi-square and it is a measure of association between the two variables; a coefficient between − 0.1 and 0.1 was considered weak/no association [19]. Analysis was performed using statistical program R version 3.5.1 [20].

**Fig. 1:**
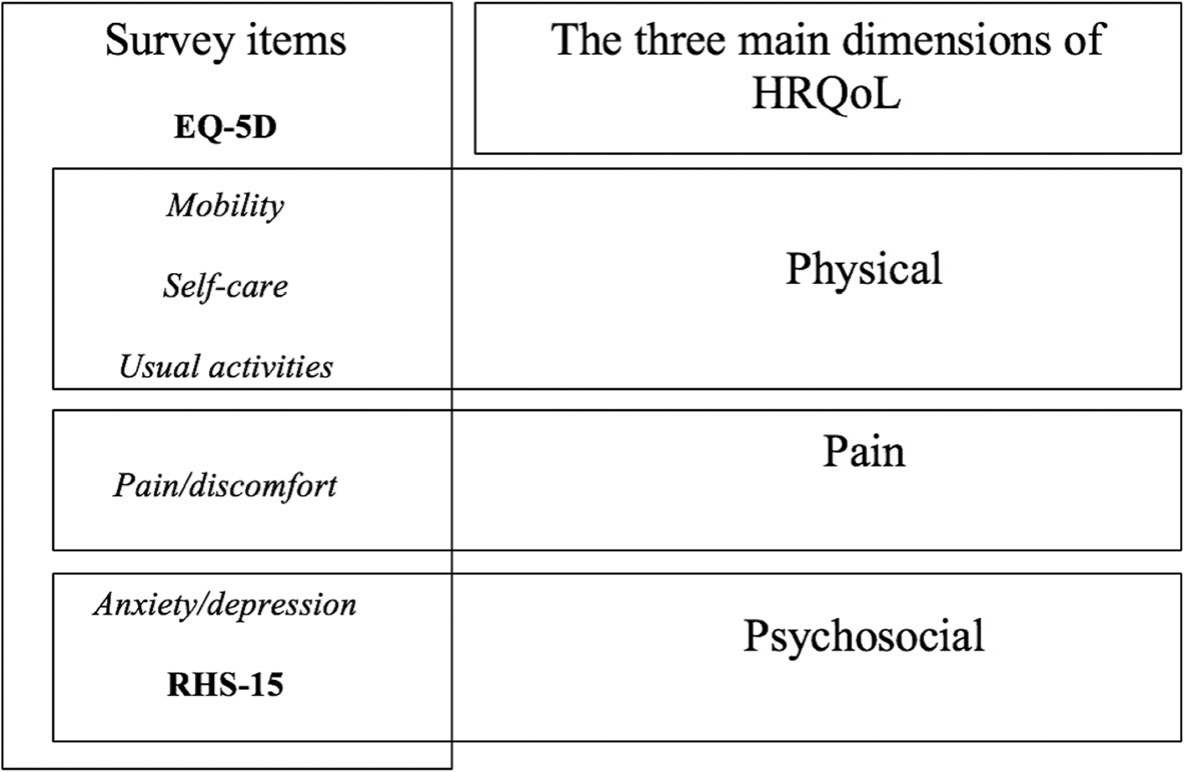
Matching the survey items to the main dimensions of HRQoL

The interviews were transcribed verbatim by JS and LJ in English and a thematic analysis approach was used for data analysis [21]. Interviews were read thoroughly by JS and LJ and thereafter coded manually. The codes were discussed with another researcher AS and sorted into emerging categories [22]. Finally, these categories were classified under the three main dimensions of HRQoL determined by Cherepenov et al. [23]: physical, psychosocial and pain. Quantitative data were also categorized using the same dimensions as shown in Fig. 1. This conceptualization of HRQoL dimensions by Cherepenov et al. [23] were used to facilitate the analysis of the data and the integration of qualitative and quantitative results.

### Mixed method integration and analysis

Integration of the survey and interviews was done first through using the quantitative data to orient the sampling of the qualitative participants, then through merging and contrasting the two datasets for comparison and presenting the results through a joint display (Table 2) [12, 14]. The joint display includes a mixed method meta-inferences that indicates whether the findings of qualitative and quantitative data confirm and reinforce each other (“confirmation”); contradict and disagree with each other (“discordance”); or whether one data set allowed expanding the understanding of the other and brought complementary aspects for the analysis (“expansion”) [14, 24].

## Results

One hundred thirty patients were identified as eligible and asked to participate in the study, 20 declined while 110 (84.6%) accepted of which 2 participants could not fill the EQ-5D survey due to language difficulties. The final sample included 108 participants, of which 104 (96%) were foreign born. Out of the 108, 95(88%) were able to complete the RHS-15 questionnaire while 13 participants did not complete this part of the survey due to language barrier. In total, 20 patients were recruited for the qualitative interviews. Baseline characteristics of all participants in the survey and qualitative interviews are summarized in Table 1.

**Table 1:** Sociodemographic characteristics of the study participants

	Quantitative survey data *n* = 108	Qualitative interview data *n* = 20
Age, mean (SD), years	29.6 (8%)	31.05 (7.8%)
Sex, n (%), women	82 (76.6%)	16 (80%)
Recent pregnancy, n (%)	59 (71.9%)	10 (62.5%)
Region of origin, n (%)		
Africa	66 (61.1%)	13 (65%)
Asia	33(30.6%)	5(25%)
Europe	8 (7.4%)	1(5%)
Other	1 (0.9%)	1(5%)
Treatment phase, n (%)		
Start (first 2 weeks)	35 (32.4%)	11(55%)
Middle	55(50.9%)	7(35%)
End (last 2 weeks)	13(12%)	2(10%)
Missing data	5(4.6%)	–

The qualitative, quantitative and mixed-method results for each of these health dimensions are presented in Table 2 and the quality of life scores results are presented in Table 3.

**Table 2:**
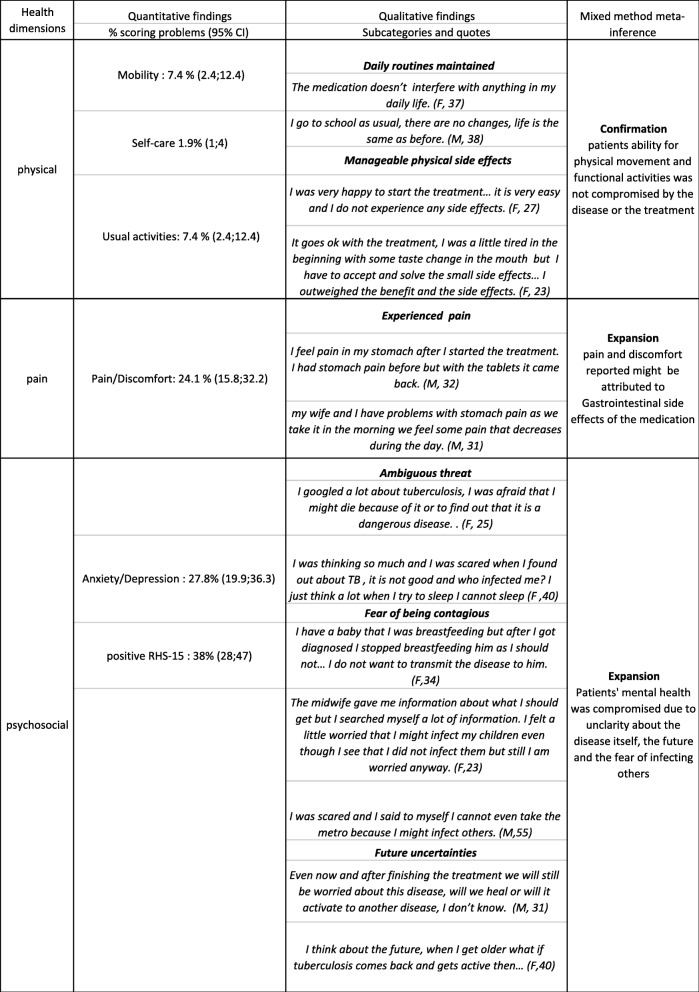
joint display of quantitative, qualitative and mixed methods inference results

**Table 3:** EQ-5D utility score and VAS scores for LTBI patients group and general population

	EQ-5D Utility score		EQ-VAS score	
	Stockholm Population	LTBI Patients	Stockholm Population	LTBI Patients
	*(n = 19,415)*	*(n = 108)*	*(n = 19,415)*	*(n = 106)*
Median	0.93	1	80	90
25th percentile	0.88	0.79	70	75
75th percentile	1	1	90	100

### Physical functioning and pain

A small percentage of patients reported problems with physical functioning in term of mobility (7.4%), and usual activity or self-care (1.8%). The qualitative interviewees highlighted that they are able to do the same activities and carry on the same routines as before the diagnosis/treatment which did not have an effect on their physical capacity. Problems with the pain/discomfort domain in the EQ-5D questionnaire were reported by 24.1% of survey participants. No/ minimal side effects were reported in the qualitative interviews except for 3 individuals who described a stomach pain/discomfort feeling associated with the medication uptake.

### Psychosocial health

Anxiety/depression is the domain with the highest percentage of problems reported as 27.8% of participants indicating some or severe problems. In addition, 36 out of the 95 participants (38%) who completed the RHS-15 screened positive on RHS-15 indicating some form of mental distress. These mental health concerns were explored further in the interviews; Interviewees mentioned that the diagnosis created some fears due to lack of knowledge and information about this type of disease and its severity, especially that TB is often associated with death and severe consequences in their home country. Patients expressed fear of being contagious and affecting their surrounding which was especially emphasized by pregnant women who were concerned about breastfeeding and being in close contact with their children. Patients also expressed their worries about activation of the latent disease in the future or “getting sick again” despite adherence to treatment.

### Quality of life

The EQ-5D utility score median was 1 with interquartile range of 0.796 and 1 while the EQ-5D VAS median was 90 with interquartile range of 75 and 100. (Table 3).

There was no statistically significant difference in EQ-5D utility score between LTBI patient and the general population (*p* = 0.079). LTBI patients had a significantly higher median EQ-VAS score than the general population (*p* = 0.032, Phi value = − 0.015).

A multilinear regression model was used to regress the EQ-5D VAS score on age, sex, recent pregnancy, country of origin and treatment phase. None of these variables were statistically significant.

## Discussion

Our results do not support the use of any HRQoL decrements for LTBI diagnosis/treatment in economic evaluations. This study found a high prevalence of positive screening results using an instrument validated for mental health screening in migrants. However, we did not compare prevalence of mental health problems with non-LTBI patients with a similar sociodemographic profile, and can therefore not draw any firm conclusion whether LTBI diagnosis in itself is associated with mental health problems. Nevertheless, these findings were supported by our mixed method analysis and may indicate that this particular patient group requires tailored psychosocial support.

EQ-5D results suggest that there is no compromise of physical function, which was also supported by the qualitative data. This finding is in line with the definition of LTBI as a condition free from physical signs or symptoms. A previous longitudinal study showed that LTBI diagnosis and treatment had a minimal HRQoL impairment compared to the control group [9].

On the other hand, the literature also emphasizes that LTBI patients may be affected by stigma and the fear of disease activation [5] which is in line with our qualitative findings. One previous study showed a decrease in the mental well-being of LTBI patients after therapy [25] which suggests that it is important to address fear and anxiety and having a referral system in place that can provide psychosocial support when needed. This approach might be particularly needed in Sweden and other low TB incidence settings where most LTBI patients are migrants, of which many are asylum seekers who may have barriers to access care and limited knowledge about the healthcare system [26, 27]. These mental health concerns were not well reflected in the overall EQ-5D scores as patients scored relatively high and very similar to the general population of Stockholm. These results bring to the forth the discussion about the heavy preponderance of physical items in EQ-5D and its questionable sensitivity to mental health concerns and detecting changes in social/psychological well-being. This generic instrument has been criticized previously on its large focus on the physical dimension of well-being compared to psychosocial dimension [28, 29].

Finally, this study along with previous literature show that fear of TB disease activation or transmission are common. While this can be considered a negative effect of a LTBI diagnosis, it may also be a motivating factor for accepting treatment. The fear and anxiety accompanying the diagnosis may thus be regarded as rational and a necessary consequence since the associated risk of progression to active TB disease indeed means a risk of severe suffering and transmission to others. Moreover, LTBI patient may not be able to clearly distinguish between the active and latent form of TB. Regardless of whether such anxiety is accounted for as a quantifiable HRQoL decrement or not, clear health information should be reinforced, clarifying the difference between TB disease and LTBI, the possibilities of disease prevention with treatment of LTBI and the consequences of not treating the condition.

In summary, better information system and a programmatic tackling of mental health concerns need to be added to LTBI management program in order to decrease the risk of fear, anxiety and stigma which directly affect the HRQoL of this patient group.

### Strengths and limitations

This study employed a mixed method design that allowed a deeper understanding of the HRQoL of a patient group using quantitative and qualitative data with matching characteristics of the two samples, rather than relying on one single approach. Using validated instruments available in the languages of the target group was another strength of this study. Despite using professional translators, some of the information might has been lost during the process of translating. Some caution is warranted when generalizing our results to other settings with a different patient profile and different practices of screening.

## Conclusion

This study supports the hypothesis of no quality of life decrement during LTBI treatment.

However, mental health concerns are common among LTBI patient in Stockholm. the signs of mental distress can be prevented through a good information system for diagnosed patients and through programmatic tackling of mental health as part of LTBI management.

## Data Availability

Not applicable. The data will not be shared as ethics approval for the study requires that the data would not locked and cannot be accessed by someone other than the researchers of the project.

## Abbreviations

HRQoL: Health-related quality of life
LTBI: Latent tuberculosis infection
RHS-15: Refugee health screener-15
TB: Tuberculosis
VAS: Visual analogue scale
WHO: World Health Organization

## References

[1] World Health organisation (2018). Latent tuberculosis infection - Updated and consolidated guidelines for programmatic management.

[2] Kim HW, Kim JS (2018). Treatment of latent tuberculosis infection and its clinical efficacy why treatment of LTBI is important. Tuberc Respir Dis (Seoul).

[3] Lonnroth K, Mor Z, Erkens C, Bruchfeld J, Nathavitharana RR, van der Werf MJ (2017). Tuberculosis in migrants in low-incidence countries: epidemiology and intervention entry points. Int J Tuberc Lung Dis.

[4] Swedish Public health agency. Recommendations for preventive measures against tuberculosis. Swedish public health agency: Stockholm; 2017.

[5] Guo N, Marra F, Marra CA (2009). Measuring health-related quality of life in tuberculosis: a systematic review. Health Qual Life Outcomes.

[6] Newbold KB, Danforth J (2003). Health status and Canada ’ s immigrant population. Soc Sci Med.

[7] Wu Z, Penning M, Schimmele CM. Immigrant status and unmet health care needs. Can J public Heal. 2005;96:369–73. 10.1007/BF03404035 PMC6975792 16238157

[8] Hansel NN, Wu AW, Chang B, Diette GB (2004). Quality of life in tuberculosis: patient and provider perspectives. Qual Life Res.

[9] Bauer M, Ahmed S, Benedetti A, Greenaway C, Lalli M, Leavens A (2015). Health-related quality of life and tuberculosis : a longitudinal cohort study. Health Qual Life Outcomes.

[10] Brown J, Capocci S, Smith C, Morris S, Abubakar I, Lipman M (2015). Health status and quality of life in tuberculosis. Int J Infect Dis.

[11] Meissner H, Creswell J, Klassen AC, Plano V, Smith KC. Best Practices for Mixed Methods Research in the Health Sciences Methods. In National Institites of Health; 2011. p. 1–39.

[12] Curry LA, Krumholz HM, O’Cathain A, Clark VLP, Cherlin E, Bradley EH (2013). Mixed methods in biomedical and health services research. Circ Cardiovasc Qual Outcomes.

[13] Wheeldon J, Ahlberg MK. Mapping mixed-methods research: theories, models and measures. Vis Soc Sci Res. 2012;4:113–48.

[14] Fetters MD, Curry LA, Creswell JW (2013). Achieving integration in mixed methods designs - Principles and practices. Health Serv Res.

[15] Martínez-Mesa J, González-Chica DA, Duquia RP, Bonamigo RR, Bastos JL (2016). Sampling: how to select participants in my research study?. An Bras Dermatol.

[16] Rabin R, De Charro F (2001). EQ-5D: a measure of health status from the EuroQol group. Ann Med.

[17] Hollifield M, Verbillis-Kolp S, Farmer B, Toolson EC, Woldehaimanot T, Yamazaki J (2013). The refugee health Screener-15 (RHS-15): development and validation of an instrument for anxiety, depression, and PTSD in refugees. Gen Hosp Psychiatry.

[18] Stockholm County Council. Hälsa Stockholm 2014. Stockholm county council: Stockholm; 2014.

[19] Murphy K, Myors B. Statistical Power Analysis: A Simple and General Model for Traditional and Modern Hypothesis Tests. New Jersey: Lawrence Erlbaum Associates; 1998.

[20] R Core Team (2018). R: a language and environment for statistical computing.

[21] Braun V, Clarke V (2008). Using thematic analysis in psychology using thematic analysis in psychology. Qual Res Psychol.

[22] Nowell LS, Norris JM, White DE, Moules NJ (2017). Thematic analysis: striving to meet the trustworthiness criteria. Int J Qual Methods.

[23] Cherepanov D, Palta M, Fryback D (2013). Underlying dimensions of the five health related quality of life measures used in utility assessment. Med Care.

[24] Guetterman TC, Fetters MD, Creswell JW (2015). Integrating quantitative and qualitative results in health science mixed methods research through joint displays. Ann Fam Med.

[25] Marra CA, Colley L, Moadebi S, Elwood RK, Fitzgerald JM (2008). Health-related quality of life trajectories among adults with tuberculosis *. Chest.

[26] Delilovic S, Kulane A, Åsbring N, Marttila A, Lönnroth K. What value for whom ? – provider perspectives on health examinations for asylum seekers in Stockholm, Sweden. BMC Health Serv Res. 2018;18:1–9. 10.1186/s12913-018-3422-1 PMC6091028 30075782

[27] Shedrawy J, Lönnroth K, Kulane A. ‘Valuable but incomplete!’ A qualitative study about migrants’ perspective on health examinations in Stockholm. Int Health. 2018. 10.1093/inthealth/ihy007. 10.1093/inthealth/ihy007 29474639

[28] Brazier J, Ratcliffe J, Salomon J, Tsuchiya A. Measuring and Valuing Health Benefits for Economic Evaluation. 2nd ed. Oxford: Oxford University Press (maker); 2016.

[29] Van De Willige G, Wiersma D, Nienhuis FJ, Jenner JA (2005). Changes in quality of life in chronic psychiatric patients : a comparison between EuroQol ( EQ-5D ) and WHOQoL. Qual Life Res.

